# Optimal spike pattern v.s. noise separation by neurons equipped with STDP

**DOI:** 10.1186/1471-2202-14-S1-P142

**Published:** 2013-07-08

**Authors:** Timothée Masquelier, Matthieu Gilson

**Affiliations:** 1 Laboratory of Neurobiology of Adaptive Process, UMR 7102, CNRS - University Pierre et Marie Curie, Paris, France; 2 Unit for Brain and Cognition, DTIC, University Pompeu Fabra, Barcelona, Spain

## 

Using numerical simulations and analytical calculations, we have recently demonstrated that, thanks to the physiological learning mechanism referred to as Spike Timing-Dependent Plasticity (STDP), neurons can detect and learn repeating spike patterns, in an unsupervised manner, even when those patterns are embedded in noise[1-3] - a computationally difficult problem. Here, we show that the learning rule is optimal, in that it maximizes the response to the patterns, while minimizing the mean response to noise. Therefore, by thresholding the response, one can at the same time maximize the hit rate, and minimize the false alarm rate.

More formally, if one considers a linear neuron, with *n *excitatory afferents, with synaptic weights *w_1 _... w_n _*in 0[1], and one input spike pattern, the goal is to find a set of synaptic weights that maximizes the neuron's response to the pattern, while minimizing the mean response to Poisson input noise. We first show that, to find the optimal set, one should convolve the input spike pattern with the excitatory postsynaptic potential (EPSP) response kernel, and select the highest resulting peaks. For each peak *p*, only a subset of the afferents *n_p _*contributed significantly to the response. One should select the highest peak with minimal *n_p_*, choose *w *= 1 for the contributing afferents, and *w *= 0 for all the others (in order to minimize the mean response to Poisson noise). Then, we investigate the conditions under which STDP indeed reaches this optimum, using analytical calculations with a linear inhomogeneous Poisson model[3,4], as well as simulations with both leaky-integrate-and-fire (LIF) and Poisson neurons.

Our results indicate that, in a number of cases, STDP indeed reaches this optimum, especially when coupled with homeostatic mechanisms. In other words, when faced with one repeating pattern to learn, STDP tends to chose the best "signature" of this pattern, that is a time window with as many (nearly) coincident spikes as possible from as few afferents as possible, and concentrates weights on these afferents only, thereby minimizing the probability of strong responses due to fortuitous spike coincidences. When faced with multiple repeating patterns, the ones with best signatures tend to be learned in priority.
